# A Practical Guide to the Newer Remedies

**Published:** 1911-06

**Authors:** 


**A Practical Guide to the Newer Remedies.** By J. M. Fortescue-Brickdale, M.A., M.D. Pp. viii., 273. Bristol: John Wright & Sons Ltd. 1910.

The necessity for a book upon the newer remedies, written by a medical man without trace of proprietary or pecuniary bias, is quite obvious to medical men. Then why has not some such book appeared before ? Undoubtedly because of the numerous and great difficulties that the author has to face at every point. At the outset there is the difficulty of selecting an arrangement that will allow of the drugs, so numerous and so diverse, falling into logical grouping. The author has to decide what constitutes a new drug, to decide the inclusions and exclusions, to weigh the worth of the pharmacological and clinical reports upon these drugs ; and in no branch of medical literature has so much been written that is weighty and valuable in appearance and so worthless and misleading in fact as on the pharmacology and therapeutics of the synthetic newer remedies. We must say at once that he has surmounted these difficulties in a wholly admirable way. Those who know this author's book, written in collaboration with Professor Francis, *The Chemical Basis of Pharmacology*, will recognise that he is pre-eminently fitted to deal with the new synthetic drugs. In this volume, however, he has wisely followed the classification of the most recent textbooks on pharmacology, rather than a purely chemical basis, with the result that his book forms an invaluable appendix to these textbooks.

The literature upon this subject is enormous, and widely scattered over medical journals, theses and so on, and the labour of sifting and condensing this literature must have occupied a very large amount of time ; yet in spite of the enormous concentration to which the material has been subjected, Dr. Fortescue-Brickdale has given us a book which is delightfully readable—indeed, many will find that they continue to read long after they have secured the particular details for which they consulted the book.

This is no mere catalogue of proprietary preparations, with extracts from suspiciously favourable and enthusiastic articles, but the author has added useful introductions to certain chapters, and shown clearly the positions of the drugs in therapeutics. The book is a valuable addition to the bookshelves of the medical practitioner, and will take its place at once amongst those volumes to which frequent reference is made. Dr. Fortescue-Brickdale trusts that this book " will be a frequent if humble adviser in the hands of some of his junior professional brethren." We believe it will have a large sale.

The author refrains from giving his own personal experience of any of these newer remedies, and at times we feel some disappointment in not finding a more definite pronouncement on the value of a drug ; but his attitude is scientifically correct, and fully justified.

There are few adverse criticisms to be made, and of these the majority depend upon facts for which the author is not responsible. But he is responsible for attributing to the *B. P. Codex* the power of giving a drug an official name (p. 96). This power rests only in the *B. P.*, for this country at any rate. No doubt the slip was due to confusion between British Pharmacopœia and British Pharmaceutical. Dr. Fortescue-Brickdale's classification of hypnotics has little advantage over a purely chemical one to which we are accustomed, and it has certain disadvantages. The division is into five groups: those which are dangerous, those presenting no advantages, mild hypnotics, medium hypnotics and powerful hypnotics. The literature on the relative "power" of the newer hypnotics is so meagre that such classification is not justified, and moreover the grouping of certain drugs as definitely dangerous implies a guarantee that those not included in that group are not definitely dangerous, a conclusion by no means justified as yet in regard to some of those not included under that damning heading.

The Introduction, unlike the vast majority of Introductions, is one of the most valuable chapters in the book, for in it the author urges a broad view of therapeutics, and adopts a sound and liberal attitude towards the newer remedies. However much a practitioner may have read about the older remedies, gradually he comes to base his practice upon his experience derived from careful observation of his own daily experiments, and in precisely the same way he must learn from personal experiment with the newer remedies. Dr. Fortescue-Brickdale has given us an efficient and reliable guide to first steps in experimenting with these drugs. As lecturer on Pharmacology to the University of Oxford, his remarks in this Introduction upon the teaching of pharmacology, and the unfortunate position allotted to it in the medical curriculum, come with weight and deserve careful consideration.

The size of the book is handy, and we do not need to say more than that the "get up" is fully up to that high standard which Messrs. John Wright and Sons have accustomed us to expect.

